# Electrospun sandwich mesh structures loaded with naringenin and vitamin K_2_ polycaprolactone/gelatin nanofibers synergistically promote bone regeneration

**DOI:** 10.1016/j.mtbio.2023.100794

**Published:** 2023-09-15

**Authors:** Jiafeng Wang, Longhui Shao, Xiaoyu Wu, Chun Liu, Su Ni, Ting Dai, Hongwei Liu, Hongbin Zhao

**Affiliations:** a Department of Orthopedics, The Affiliated Changzhou Second People's Hospital of Nanjing Medical University, Changzhou, 213164, China; b Dalian Medical University, Dalian, 116044, China

**Keywords:** High-voltage electrospinning, Naringenin, Vitamin K_2_, Nanofibers, Bone regeneration and repair

## Abstract

Osteoblasts and osteoclasts play a crucial role in the dynamically coupled balance during bone regeneration and remodeling. They complement and restrict each other in the human body. Decreased osteoblasts lead to insufficient bone formation or excessive formation of osteoclasts, leading to increased bone resorption, which will destroy the structure of the bone tissue. This will greatly increase the risk of diseases such as osteoporosis and nonunions caused by bone defects.

Herein, gelatin and polycaprolactone were used as substrates, and biomaterial membranes with mesh and sandwich structures were constructed using the electrospinning technology. Naringenin was loaded into the shell, and vitamin K_2_ was loaded into the core layer of the nanofibrous membrane. The biocompatibility and osteogenic capacity of the membranes were assessed in vitro using mouse bone marrow mesenchymal stem cells (BMSCs). During osteoclast induction, the receptor activator of nuclear factor kappa-Β ligand (RANKL) was used to coculture RAW264.7 cells with various materials. The regulatory effect of various membranes on osteoclast growth was evaluated by detecting the expression levels of osteoclast-related genes and proteins in the cells.

Subsequently, we constructed a model of a rat skull defect and implanted different membranes into the defect. Then, we evaluated the new bone formation in the defect using histological staining and micro-computed tomography after 4 and 8 weeks. The results of in vitro experiments confirmed that the incorporation of naringenin and vitamin K_2_ stimulated the expression of osteogenesis-related genes and the secretion of osteogenesis-related proteins. Simultaneously, the results showed that naringenin and vitamin K_2_ inhibited the formation and growth of osteoclasts. Therefore, naringenin and vitamin K_2_ have a synergistic effect in promoting bone growth and regulating osteoclast growth.

## Graphical abstract

**Figure:**
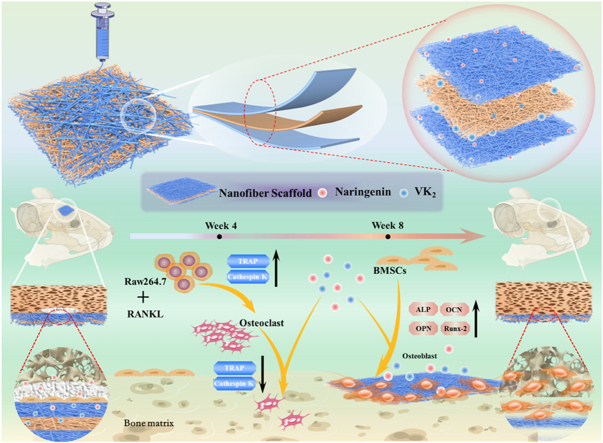

## 1 Introduction

Bones are the hard organs of the body and play an important role in support, protection, and movement. Their unique and powerful regenerative abilities can repair minor bone damage. However, in complex cases, such as osteoporosis, tumors, infections, and severe trauma, the bone healing process is often disturbed, resulting in delayed union or even nonunion of bone defects, which has become a major problem in the orthopedic field [1,2]. The treatment of bone defects mainly includes autologous and allogeneic bone grafts and bone tissue engineering [3,4]. Autologous bone grafting is currently the most widely used clinical method, and it is also considered the gold standard in bone defect treatment. However, this procedure causes secondary injury to the patient, increases the risk of infection, and limits clinical application due to the limited source of autologous bone [5,6]. Therefore, a more advanced treatment approach is needed to solve severe bone defects. In this context, electrospinning, three-dimensional (3D) printing, and in vitro cell culture technologies have been applied to bone tissue engineering, and great progress has been made. Bone tissue engineering is considered a promising alternative to traditional bone grafting [7]. After years of development, artificial bone repair materials have evolved from bioinert materials without the osteoinductive ability to smart materials with osteoconductive and osteoinductive effects, suitable porosity, excellent mechanical properties, and degradability [8,9].

In recent years, with the rapid development of bone tissue engineering and nanotechnology, electrospinning technology has occupied an important position. The fiber membrane prepared by electrospinning technology has the characteristics of high porosity and nano-scale morphology, which has similar advantages to natural extracellular matrix, and can promote cell adhesion and growth and other physiological processes. Nanofibrous scaffolds prepared by electrospinning are often used as drug carriers because of their slow-release effects. More complex electrospinning structures are used for drug loading, such as coaxial electrospinning, triaxial electrospinning, and sandwich-like multi-layered electrospinning scaffolds, the role of which is to change the position of the drug in the scaffold, the distribution changes the release rate of the drug, which can better control the release rate of the drug, increase the utilization rate of the drug, and reduce the side effects of the drug [10,11].

Polycaprolactone (PCL) is a synthetic polymer with excellent biocompatibility and mechanical properties. Simple PCL scaffolds are not very hydrophilic, are slow to degrade, and lack biological activity, which limit their application in bone tissue engineering [12,13]. Gelatin is a denatured collagen product with low antigenicity, good hydrophilicity, and the ability to promote cell adhesion. However, gelatin scaffold alone has insufficient mechanical properties, poor thermal stability, and excessive degradation rate, which limit its use in bone tissue engineering [14,15].

Naringenin, a flavonoid found in citrus fruits and grape skins, has anti-inflammatory and antioxidant properties and excellent therapeutic properties in the treatment of osteoporosis, cardiovascular disease, and cancer [[16], [17], [18]]. Recent studies have also shown that naringenin exhibits estrogen-like effects, can effectively promote the transformation of mesenchymal stem cells into osteoblasts, and can improve the ability of cell mineralization [19].

Vitamin K_2_ is a naphthoquinone compound with an isoprene side chain [20]. In addition to its well-known function of supporting blood clotting protein synthesis, it plays a vital role in maintaining heart, bone, and blood vessel health [21]. Vitamin K_2_ also promotes the growth and differentiation of osteoblasts and bone calcium deposition. In terms of antiosteoporosis, it can reduce the activity of osteoclasts and prevent bone loss [[22], [23], [24]].

Herein, we used electrospinning technology to fabricate nanofibrous scaffolds with mesh and sandwich structures using polycaprolactone and gelatin as substrates. Simultaneously to loading naringenin into the shell of the scaffold, vitamin K_2_ was loaded into the core layer of the scaffold. The effects of naringenin and vitamin K_2_ on promoting osteogenesis and regulating osteoclast growth were evaluated in vitro and in vivo.

## 2 Materials and methods

### 2.1 Fabrication of nanofibrous membranes

Fiber membrane core layer preparation: For the preparation, 1.2 g of PCL (Sigma-Aldrich, USA) and 1.2 g of gelatin (∼250 g Bloom, Aladdin, China) were dissolved in 24 mL of hexafluoroisopropanol. The solution was stirred with a magnetic stirrer for 6 h at room temperature, 1 × 10^−3^ moL of Vitamin K_2_ (Aladdin, China) was added, and the solution was stirred until it is uniform.

Fiber membrane shell layer preparation: For the preparation, 2.88 g of PCL was dissolved in 24 mL of hexafluoroisopropanol and stirred at room temperature for 6 h; after it was completely mixed, 1 × 10^−2^ moL of naringenin (purity ≥97%; Aladdin, China) was added, and the solution was stirred again to a homogeneous solution. The prepared composite solution was then subjected to electrospinning. First, a copper mesh was laid on the capture roller of the electrospinning device. Subsequently, 12 mL of shell layer liquid was put into the syringe; the parameters were set as follows: voltage 14 kV, feed speed 0.0032 mm/s, drum speed 40 rpm, the distance between the needle tip and the drum 12 cm, humidity 50% in the electrospinning machine, and temperature 28 °C. After spraying, 24 mL of core layer solution was collected. The parameters were reset as follows: voltage 16 kV, propulsion speed 0.0032 mm/s, barrel speed 40 rpm, and the distance between the needle tip and barrel 10 cm, with the temperature and humidity kept constant. After the 24-mL solution was sprayed, it was switched back to the shell layer solution, and 12 mL was sprayed. Finally, the nanofibrous film was carefully peeled off the copper grid and placed in an oven at 37 °C overnight. According to which drugs were added to the above two solutions, four kinds of material scaffolds were finally prepared, namely, the PG group (without drug), the PG-N group (only containing naringenin), the PG-V group (only containing vitamin K_2_), and the PG-NV group (containing naringenin and vitamin K_2_).

### 2.2 Morphology and characterization of the nanofibrous membrane

The microstructure of nanofibrous membranes was observed using scanning electron microscopy (SEM) (JSM-IT510 In TouchScope, Japan). First, the membrane was freeze-dried under vacuum overnight, then the sample was placed under an argon atmosphere, and a layer of platinum was sprayed. The morphology and structure of the frame were observed under a 100 × and 1000 × scanning electron microscope, and the fiber scaffold was observed under an optical microscope.

### 2.3 Membrane hydrophilicity detection

The water contact angle of the fiber membranes was measured using an OCA-20 contact angle meter. A 0.5 × 0.5 cm holder was placed on the detection platform, and the instrument was controlled to drop 5 μL of distilled water onto the surface of the membrane. The image of the water contact angle formed between the membrane and the water droplet was recorded.

### 2.4 Air permeability test

First, 5 mL of distilled water was added to a small glass bottle without a cap. The fibrous membrane was then cut into a circle large enough to cover the mouth of the bottle, and the measured total mass was M1. The flask was placed in a 37 °C environment for 24 h, and the mass was measured again as M2. Simultaneously, a control group was set up (the bottle mouth was not sealed with a fiber film), the measured mass was M3, and the measured mass after 24 h in the incubator was M4. The calculation formula for air permeability was as follows:

$$\text{Air}\;\text{permeability}\;\left(\%\right)=\frac{M1-M2}{M3-M4}\times 100\%$$

### 2.5 Water absorption test

After immersing the dried membrane in pure water at 37 °C for 24 h, the water on the surface of the membrane was removed, the changes in the mass of the membrane before and after weighing were weighed, and the water absorption performance of the microscopic membrane was measured. The formula for membrane water absorption is as follows:

$$\text{Swelling}\;\left(\%\right)=\frac{M2-M1}{M1}\times 100\%$$

where *M*1 is the dry weight of the membrane, and *M*2 is the mass of the membrane after absorbing water.

### 2.6 Determination of porosity

First, 5 mL of absolute ethanol was added to a graduated cylinder, and the volume *V*1 was recorded. The nanofibrous membrane was completely immersed in ethanol, and the total volume was measured as *V*2. Subsequently, the nanofibrous membrane was removed from the ethanol, and the remaining ethanol volume was measured as *V*3. The formula for calculating porosity is as follows:

$$\text{Porosity}\;\left(\%\right)=\frac{V1-V3}{V2-V3}\times 100\%$$

### 2.7 FTIR and Raman spectroscopic analysis

The functional group composition of the nanofibrous material was detected using Fourier transform infrared spectroscopy (FTIR, PerkinElmer 1600 series, USA). The nanofiber membrane was cut into 1 × 1 cm (length × width) and put in the spectrometer chamber to obtain the spectral data from 4000 cm^−1^–400 cm^−1^. At the same time, we also conducted Raman spectroscopy (Rennishaw inVia, Britain) detection on the nanofiber film. The wavelength of the laser light source was 633 nm, the laser power was 2.5 mW, the grating was 600 gr·mm^−1^, and the scanning time was 10 s. The spectral data of 50–3000 cm^−1^.

### 2.8 In vitro drug release

The effect of releasing drugs of the nanofibrous membrane was tested. The scaffolds of the PG-N and PG-V groups were cut into 2 × 2 cm (length × width) pieces, put in a centrifuge tube filled with 50 mL of phosphate-buffered saline (PBS) (0.1 M, pH = 7.4), and shaken well at 37 °C. Subsequently, 1 mL samples were collected at 2 h, 4 h, 12 h, 24 h, 2 d, 3 d, 4 d, 5 d, 6 d, 7 d, and 14 d, and the drug concentration was detected using a spectrophotometer (Shimadzu, Japan). The formula for calculating the cumulative release of naringenin and vitamin K_2_ is as follows:

$$\text{Cumulative}\;\text{release}\;\left(\%\right)=\frac{CnV0+V1\sum C\left(n-1\right)}{w}\times 100\%$$

where *Cn* represents the concentration of the 1 mL sample, *V*0 represents the volume of PBS in the centrifuge tube (50 mL), *V*1 represents the volume of each sampling (1 mL), *n* represents the number of sampling times, and *w* represents the content of naringenin or vitamin K2 in the total membrane mass.

### 2.9 In vitro degradation evaluation

The sample was lyophilized under vacuum, and the measured mass was *M*1. Then, the membranes were immersed in 40 mL of 37 °C PBS and removed on days 3, 7, 14, 21, and 28. The scaffold was rinsed with distilled water and dried under vacuum, and the membrane weight was measured as *M*2. The degradation rate is calculated as follows:

$$\text{Degradation}\;\text{rate}\;\left(\%\right)=\frac{M2-M1}{M1}\times 100\%$$

### 2.10 Mechanical property testing

The nanofiber membrane was cut into a 1 × 5 cm rectangle, and the tensile properties of fiber membranes were measured using a tensile testing machine (MTS system Co., LTD, China) in a dry state. Specifically, the two ends of the nanofiber membrane were clamped by the instrument, and the fiber membrane was stretched at a speed of 3 mm per min until the fiber membrane broke. Finally, a stress–strain curve was obtained.

### 2.11 In vitro cytocompatibility and proliferation assays

#### 2.11.1 Cell culture and preparation of extracts

Mouse bone marrow mesenchymal stem cells (BMSCs; CRL-12424, ATCC, USA) were cultured in Dulbecco's-modified Eagle's medium (DMEM basic, Gibco, Thermo Fisher Biochemical Products, USA) containing 10% fetal bovine serum (Biological Industries, Israel) and 1% penicillin/streptomycin (Life Technologies Corporation, USA) in a 37 °C, 5% CO_2_ environment. Membrane extracts were prepared by immersing the membrane in complete DMEM for 1 d, passing through a filter, and storing at 4 °C according to the national standard of China (GB/T 169886.12).

#### 2.11.2 Live/dead cell and phalloidin staining experiments

The toxicity of nanofibrous membranes was evaluated using a Calcein/PI Cell Viability and Cytotoxicity Assay Kit (Beyotime, China). After the extracts of each group were cultured with BMSCs for 3 d, Calcein/PI working solution was added to each well, incubated in the dark for 30 min, and observed under a fluorescence microscope (Olympus, Japan). Furthermore, we observed the effects of naringenin and vitamin K_2_ on the cytoskeleton morphology using a phalloidin reagent. BMSCs were cultured with the membrane for 3 d, and then the cells were fixed with 4% paraformaldehyde, infiltrated with 0.5% Triton X-100 solution, covered with the prepared FITC-labeled phalloidin working solution, and incubated in the dark for 30 min. The nuclei were stained with DAPI (Dojindo Laboratories, Kumamoto, Japan), and the cell morphology was photographed under a fluorescence microscope (Olympus, Japan).

#### 2.11.3 BMSC adhesion assay

BMSCs were seeded onto 2 × 2 cm nanofiber membranes and cocultured for 3 d (1 × 10^5^ cells per well). The cells were fixed using glutaraldehyde (2.5%). The fiber membrane was soaked and dehydrated with different concentrations of alcohol (60%, 70%, 80%, 90%, and 100%) and then vacuum dried. Finally, the cell morphology and number on the fibrous membrane surface were observed using a scanning electron microscope.

#### 2.11.4 Cell proliferation assay

The effect of different nanomembranes on the proliferation of BMSCs was evaluated using a cell counting kit 8 (CCK-8; Dojindo Kagaku, Japan). BMSCs were seeded into 96-well plates at a density of 3 × 10^3^ cells per well and cocultured with membrane extracts for 1, 3, and 7 d. CCK-8 was added at each time point and incubated for 1.5 h in the dark. Finally, the absorbance of each membrane was detected at 450 nm using a microplate reader.

### 2.12 Osteogenic capacity evaluation of fibrous membranes

#### 2.12.1 Expressions of osteogenic genes

The expression levels of osteogenic genes in BMSCs in each group were detected using the reverse transcription-quantitative polymerase chain reaction (RT-qPCR) to evaluate the osteogenic ability of membranes in vitro. Specifically, BMSCs were cocultured on fiber membranes with a cell number of 8 × 10^4^ cells per well in a 6-well plate. On days 3 and 7, cellular RNA was extracted using the NucleoZol kit (Macherey-Nagel, Germany). Then, the HiScript II Q RT SuperMix kit (Vazyme, China) was used to convert equal amounts of RNA into cDNA. The reverse transcription was performed according to the manufacturer's instructions, and gene amplification was then performed using a SYBR Green Master Mix kit (Vazyme, China) and osteogenesis-related primers. Primers for Osteogenesis-related genes are listed in Table 1.

**Table 1:** Osteogenesis-related primer sequences.

Gene	Primer	Primer sequences
GAPDH	Forward	5′-CACCACCAACTGCTTAGC-3′
	Reverse	5′-TTCACCACCTTCTTGATGTC-3′
OSX	Forward	5′-ATCGAGTACATCTTCAAGCCAT-3′
	Reverse	5′-GRGAGGTTTGATCCGCATAATC-3′
RUNX-2	Forward	5′-CTGCAAGCAGTATTTACAACAGAGG-3′
	Reverse	5′-GGCTCACGTCGCTCATCTT-3′
ALP	Forward	5′-GCAGTATGAATTGAATCGGAACAAC-3′
	Reverse	5′-ATGGCCTGGTCCATCTCCAC-3′
COL-1	Forward	5′-GACATGTTCAGCTTTGTGGACCTC-3′
	Reverse	5′- GGGACCCTTAGGCCATTGTGTA-3′
OCN	Forward	5′-ACCATCTTTCTGCTCACTCTGCT-3′
	Reverse	5′-CCTTATTGCCCTCCTGCTTG-3′
OPN	Forward	5′-TACGACCATGAGATTGGCAGTGA-3′
	Reverse	5′-TATAGGATCTGGGTGCAGGCTGTAA-3′

#### 2.12.2 Alkaline phosphatase and alizarin red S staining

BMSCs were cultured in 6-well plates (4 × 10^4^ cells per well) with the membrane. After 7 d, BCIP/NBT staining solution (Beyotime, China) was added and the cells were incubated in the dark for 3 h at 37 °C. The alkaline phosphatase activity of each group was observed with an optical microscope. Similarly, after 14 d of coculture of BMSCs and membranes, alizarin red S staining reagent was added and the cells and membranes were incubated in the dark for 1 h. The deposition of calcium nodules in each group was observed under a microscope.

#### 2.12.3 Western blotting experiment

BMSCs were seeded in 6-well plates (8 × 10^4^ cells per well) and cultured for 3 d. Total protein was extracted with RIPA tissue lysate supplemented with protein phosphatase and protease inhibitors and SDS-PAGE protein loading buffer. SDS-PAGE gel (Vazyme, China) electrophoresis was used to separate proteins with different molecular weights, and then the protein bands were transferred onto polyvinylidene fluoride (PVDF) membranes (Solarbio, China). After soaking in 5% skimmed milk for 1.5 h, the membrane was immersed in *anti*-RUNX-2 (1:2000 dilution, Proteintech, China), anti-COL-1 (1:2000 dilution, Proteintech, China), *anti*-OPN (1:800 dilution, Santa Cruz, USA), or anti-ALP (1:800 dilution, Santa Cruz, USA) primary antibody, overnight at 4 °C. The PVDF membrane was washed with TBST (1000 mL TBS +1 mLTween 20) solution and immersed in antirabbit or antimouse secondary antibody (1:1000 dilution, Proteintech, China) for 1 h. ECL luminescent liquid (NCM Biotech, China) was used for color development, and the levels of osteogenesis-related factors secreted by BMSCs in each group were evaluated.

#### 2.12.4 Immunofluorescence staining

BMSCs were seeded in 24-well plates (3 × 10^3^ cells per well). After 3 d of coculture with the extracts, the cells were fixed with 4% paraformaldehyde, and the cell membrane was permeabilized with 0.2% Triton X-100 and blocked with 5% bovine serum albumin (BSA, Beyotime, China). Subsequently, the cells were incubated with a specific primary antibody (RUNX-2, 1:50 dilution, Santa Cruz, USA; OPN, 1:50 dilution, Santa Cruz, USA) at room temperature for 2 h and then incubated with an antimouse/rabbit secondary antibody conjugated to fluorescent dyes (1: 500, Abcam, Britain) for 1 h in the dark. Finally, cells were counterstained with DAPI solution (10 μM) and observed under a fluorescent microscope.

### 2.13 RAW264.7 cell culture and osteoclast induction

RAW264.7 cell (cell research, China) culture: DMEM containing 10% fetal bovine serum and 1% penicillin/streptomycin was used. The culture conditions were 37 °C, 5% CO_2_, and humidity of 70%–80%.

Osteoclast induction: RAW264.7 cells were seeded in a 6-well plate (1.8 × 10^5^ cells per well), and the experimental group was given a receptor activator of nuclear factor kappa-Β ligand (RANKL)-inducing factor (Servicebio, China) at a concentration of 50 ng/mL, and the medium was replaced every 2–3 days and cultured for 5 d. Subsequently, the expressions of osteoclast-related genes before and after induction were compared to evaluate the success of osteoclast induction. Primers for osteoclast-related genes are listed in Table 2.

**Table 2:** Osteoclast-related primer sequences.

Gene	Primer	Primer sequences
GAPDH	Forward	5′-CACCACCAACTGCTTAGC-3′
	Reverse	5′-TTCACCACCTTCTTGATGTC-3′
TRAP	Forward	5′-TGCGACCATTGTTAGCCACATACG-3'′
	Reverse	5′-CACACCGTTCTCGTCCTGAAGATA-3′
Cathepsin K	Forward	5′-AGCAGAAACTTGAACACCCACATCC-3′
	Reverse	5′-ACCCTTAGTCTTCCGCTCACAGTAG-3′

### 2.14 Effects of membranes on the expressions of osteoclast-related genes

Briefly, RAW267.4 cells were seeded into 6-well plates (1.8 × 10^5^ cells per well), followed by adding RANKL and fibrous membrane. On day five, RT-qPCR detection was performed using the same method.

### 2.15 Cytoskeleton staining experiment

A phalloidin staining kit (Yeasen Biotechnology, China) was used to evaluate the effect of RANKL on the morphology of RAW264.7 cells. First, RAW264.7 cells were seeded in 24-well plates at a density of 3.75 × 10^4^ cells per well. On day five, the cells were fixed with 4% paraformaldehyde, permeabilized with 0.5% Triton X-100 solution, added FITC-labeled phalloidin working solution, and counterstained with DAPI. The cell morphology was observed under a fluorescent microscope.

### 2.16 Immunofluorescence staining

RAW264.7 cells were seeded in a 24-well plate at a cell density of 3.75 × 10^4^ cells per well. After the cells adhered, the extract was replaced, the RANKL was added, and the NC group (negative control group) was set simultaneously. After 5 d, the cells were fixed with 4% paraformaldehyde, and the cell membrane was permeabilized with 0.2% Triton-X100 and blocked with 5% BSA. Then, the cells were incubated with antitartrate-resistant acid phosphatase (TRAP) primary antibody (1:100 dilution, Servicebio, China) at room temperature for 2 h and then incubated with fluorochrome-conjugated antirabbit secondary antibody for 1 h in the dark. Finally, the nuclei were stained with DAPI solution and observed under a fluorescent microscope.

### 2.17 TRAP staining

TRAP staining (Servicebio, China) was used to evaluate the effect of different fibrous membranes on osteoclast formation. RAW264.7 cells were seeded into 12-well plates at a density of 8.5 × 10^4^ cells per well. After the cells adhered, RANKL and membrane extracts from each group were added. After 5 d, the cells were fixed with 4% paraformaldehyde and permeabilized with 0.2% Triton X-100 solution, and the TRAP working solution was added and incubated in the dark at 37 °C for 1.8 h. Finally, the cells were counterstained with hematoxylin and observed under a microscope.

### 2.18 Western blotting

RAW264.7 cells were seeded in 6-well plates (1.8 × 10^5^ cells per well). After the cells adhered, the extract containing the RANKL was replaced. After culturing for 5 d, the same method was used to extract the total protein, separate proteins, transfer the protein onto the PVDF membrane, and block. PVDF membranes were then soaked in anti-TRAP (1:1000 dilution, Servicebio, China) or *anti*-RANKL (1:1000 dilution, Servicebio, China) primary antibody overnight at 4 °C. After washing with TBST, the PVDF membranes were exposed to a diluted antirabbit secondary antibody for 1 h. Finally, the color was developed using ECL luminescent liquid.

### 2.19 Construction of a rat skull defect model and implantation of fibrous membranes

To investigate the osteogenic potential of each group of fibrous membranes in animals, we established a rat skull defect model. Rats were randomly divided into NC, PG, PG-N, PG-V, and PG-NV groups, 10 in each group. Rats were anesthetized with 3% pentobarbital (100 mg/kg) before surgery. A 2-cm skin incision was made in the center of the skull with a scalpel, the subcutaneous soft tissue was dissected to expose the skull, and an incision with a diameter of 5 mm was cut on the rat skull using electroporation. Two symmetrical circular bone defects were created on the rat. Subsequently, each group of fibrous membranes was implanted, and the wound was sutured layer by layer. At four and eight weeks postsurgery, the rats were sacrificed in batches using excessive pentobarbital, and the skulls were dissected, soaked in 10% neutral formalin, and fixed for one week. The animal experiment was approved by the Animal Committee of the Changzhou No. 2 People's Hospital ([2017] KY035-01). Animal experiments were performed in accordance with the guidelines of the Animal Care and Use Committee (IACUC).

### 2.20 Micro-CT scan and evaluation

To quantify new bone growth in bone defects, 3D images of rat skulls were acquired using a micro-CT scanner (Quantum GX, PerkinElmer, America) at 4- and 8-weeks postoperation. Bone repair parameters, such as the bone mineral density, bone volume fraction, trabecular bone count, and trabecular bone separation, were also evaluated in different groups.

### 2.21 Histology and immunohistochemical staining

Samples were immersed in 5% EDTA (pH = 7.2–7.4) for 40 d to decalcify. Then, they were dehydrated, paraffin-embedded, and sectioned to 6-μm thickness. Subsequently, hematoxylin and eosin staining (Servicebio, China), Masson's trichrome staining (Servicebio, China), and TRAP staining were performed according to the manufacturer's instructions. In addition, the expression levels of osteogenesis-related proteins in the new bone were detected using immunohistochemical staining. Sections were dewaxed, infiltrated with 1% Triton X-100, and incubated with 3% sodium hydroxide, and pepsin antigen retrieval solution and 5% BSA were added dropwise; then, the sections were soaked in primary antibodies (*anti*-OCN, *anti*-OPN, and anti-COL antibodies; 1:200 dilution; Santa Cruz, USA) overnight at 4 °C. After washing with PBS, an antimouse secondary antibody was added and incubated at 37 °C for 30 min. Finally, the DAB was used to develop the color, and the nuclei were stained with hematoxylin, washed with water for 10 min, dehydrated, and mounted with resin.

### 2.22 Statistical analysis

Data analysis was performed using GraphPad Prism 9.0 software. Results are presented as mean ± standard deviation (SD). One-way analysis of variance was used to evaluate the difference between the two groups, and *p* < 0.05 was considered statistically different.

## 3 Results

### 3.1 Morphological characterization of nanofibrous membranes

**Fig. 1:**
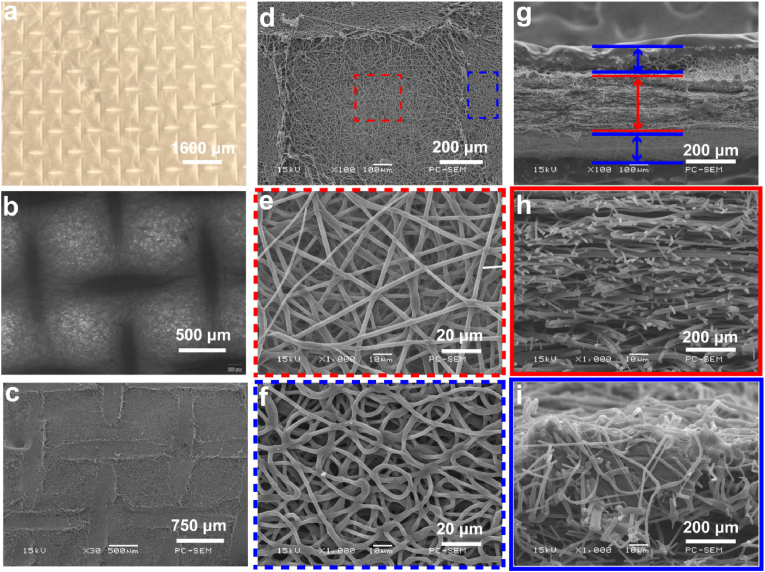
Morphological structure of the membrane. (**a**) Gross morphology of the membrane; (**b**) light microscopy image of the membrane; (**c–f**) scanning electron microscopy (SEM) image of the membrane surface; and (**g–i**) SEM image of membrane cross-section.

The morphology of the constructed nanofibrous membranes is shown in Fig. 1. Fig. 1a–c can be concluded that the surface of the constructed nanofibrous membrane has a mesh-like shape. Fig. 1d–f, it can be seen that the interlaced nanofibers are evenly distributed in the membrane, forming a loose porous structure. The nanofibers in the center of the mesh were sparsely distributed, which increased the porosity of the material. However, on the mesh line, the fiber distribution was relatively dense, where the thickness of the membrane was relatively large, which can increase the mechanical properties to a certain extent. Fig. 1g–i is the cross-sectional image of the nanofibrous membrane under the scanning electron microscope. It can be observed that the membrane structure is similar to a sandwich structure, and the fibers in the core layer are relatively loose, while the fibers in the surface layer are relatively dense. Fig. S1 is the scanning electron microscope image of the interface between the inner and outer layers. Compared with the core layer, the mesh shape of the outer layer is more obvious, the morphology is rougher, and the nanofibers are more curved. The results of element mapping are shown in Fig. S2. Naringenin and vitamin K_2_ are composed of C, H, and O elements, while gelatin and PCL are mainly composed of C, H, and O elements, and the added drug content is low, so the drug the addition of will not significantly change the elemental composition of the fiber membrane.

### 3.2 Determination of hydrophilicity, air permeability, water absorption, and porosity of nanofibrous membranes

**Fig. 2:**
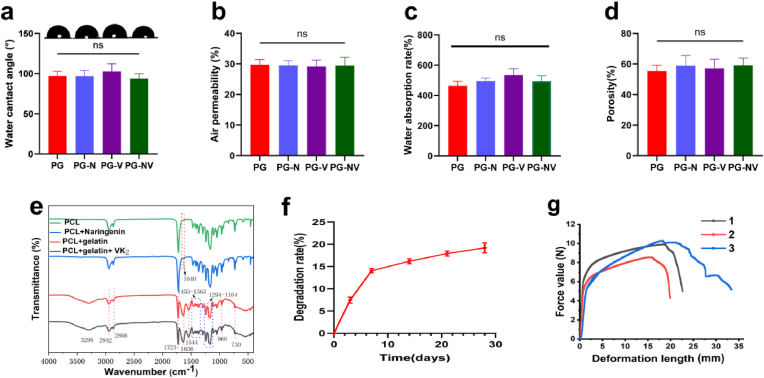
**Characterization of the nanofiber membranes.** (**a**) Hydrophilicity of the different membranes; (**b**) air permeability; (**c**) water absorption rate; (**d**) porosity of the membranes; (**e**) FTIR spectra; (**f**) cumulative release curves of naringenin and vitamin K_2_; (**f)** degradation of membranes; and (**g**) the tensile mechanical properties of membranes. 1.2.3 represents 3 parallel samples of the NC group. PG is the unloaded group, PG-N is the naringenin-loaded group, PG-V is the vitamin K_2_-loaded group, and PG-NV is the simultaneously loaded naringenin and vitamin K_2_ group. Data are presented as mean ± SD.

As shown in Fig. 2a, there was no significant difference in the hydrophilicity of the nanofibrous membranes in each group (*p* > 0.05). Adding a small amount of drug to the surface did not significantly affect the hydrophilicity of the scaffold.

As shown in Fig. 2b, there was no significant difference in the air permeability of the nanofibrous membranes in each group (*p* > 0.05). Compared with the negative control group, the air permeability was 30%, indicating that the nanofibrous membranes had good air permeability and porosity.

Fig. 2c shows the water absorption of different nanofibrous membranes. The results showed that the water absorption of the drug-loaded nanofibrous membranes was not significantly different compared with the drug-free membranes (*p* > 0.05).

Fig. 2d shows the porosity of each group of fibrous membranes. The porosity of the scaffolds in each group was between 55% and 60%, indicating that the constructed membranes had high porosity, but there was no statistical difference between the groups.

### 3.3 Infrared spectroscopy, degradation rate, and mechanical properties analysis of nanofibrous scaffolds

In order to examine the composition of the nanofibrous membrane, we carried out infrared spectroscopy on the surface and core layers of the non-drug-loaded fiber membrane and the surface and core layers of the drug-loaded membrane. The results are shown in Fig. 2e, the absorption peak of the PCL + naringenin group at 1640 cm^−1^ is caused by the vibration of the benzene ring skeleton on naringenin. The absorption peak at 1723 cm^−1^ is caused by C=O in PCL and naringenin, The absorption peak at 1636 cm^−1^ is mainly due to C=O on gelatin, and also contains the benzene ring skeleton vibration of vitamin K_2_ and the stretching vibration absorption peak of C=C. After comparison, it can be seen that the incorporation of naringenin and vitamin K_2_ does not generate new absorption peaks, and there is no chemical reaction with gelatin and PCL, but pure physical incorporation. At the same time, the nanofiber membrane was detected by Raman spectroscopy. As shown in Fig. S3a, after adding naringenin, no obvious new characteristic peaks appeared, only a weak peak change appeared at 1625 cm^−1^, which may be caused by the vibration of the benzene ring skeleton of naringenin. Fig. S3b, after adding vitamin K_2_, the Raman spectrum also has no obvious change, and there is a weak peak change at 1700-1500 cm^−1^, the result is similar to the FTIR. In addition, an increase in the intensity of some peaks in the spectrum was observed. According to the chemical structure formula of the drug and the material, we speculate that the combination of the drug and the nanofibrous membrane generates hydrogen bonds that change the vibrational behavior of the polymer chain, resulting in an increase in the intensity of the peak. It is also possible that the drug is physically adsorbed (van der Waals forces) on the material, causing interaction forces between it and the polymer chains, thereby enhancing the vibration of this functional group.

The cumulative release curves of naringenin and vitamin K_2_ are shown in Fig. 2f. The curves show that both drugs exhibit explosive release on the first day of administration, but the drug release gradually slows down and tends to stabilize 2–7 d after administration. The results showed that the nanofibers successfully released naringenin and vitamin K_2_.

Fig. 2f shows the fibrous membrane (PG group) degradation in vitro for four weeks. The degradation rate of the scaffold was relatively fast in the first week, accounting for approximately 14% of the total mass.

Fig. 2g is the test curve of the tensile mechanical properties of the fiber membranes. The average maximum tensile force the fiber membrane could withstand was 9.58 N, and the average maximum deformation was 25.36 mm.

According to the mechanical experiment results, the Young's modulus of the material is calculated to be about 47.1 MPa.

### 3.4 Sustained release of naringenin and vitamin K_2_ in vitro

**Fig. 3:**
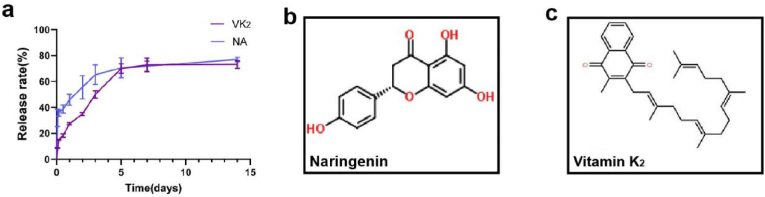
**Sustained release of drugs from nanofibrous membranes in vitro.** (**a**) Cumulative release rate of naringenin and vitamin K_2_; (**b**) The structural formula of naringenin; (**c**) The structural formula of vitamin K_2_.

The cumulative release curves of naringenin and vitamin K_2_ are shown in Fig. 3a. The curves show that both drugs exhibit explosive release on the first day of administration, but the drug release gradually slows down and tends to stabilize 2–7 d after administration. The results showed that the nanofibers successfully released naringenin and vitamin K_2_. Fig. 3b and c are the structural formula of naringenin and vitamin K_2_.

### 3.5 Biocompatibility evaluation of the membrane

**Fig. 4:**
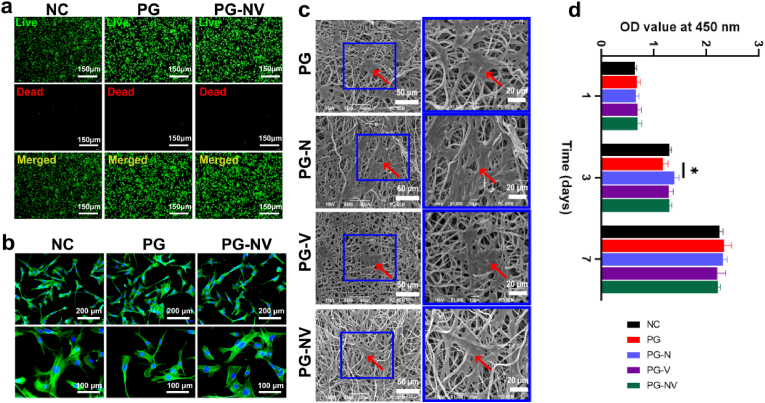
**Biocompatibility of different fiber membranes.** (**a**) Live/dead cell staining images; (**b**) cytoskeletal staining image of BMSCs; (**c**) observation of BMSCs on different membranes by SEM; and (**d**) proliferation rate of BMSCs and extracts from different groups after being cultured for 1, 3, and 7 days was determined using CCK-8. Data are presented as the mean ± SD (*n* = 3). *p < 0.05.

Good biocompatibility is a prerequisite for scaffolds. Fig. 4a shows live/dead staining images of membrane extracts cocultured with BMSCs for 3 d for each group. After the cells were cocultured with the membrane extract, only a few cells died, and most of the cells survived and grew normally, proving that the cytotoxicity of the membranes in each group was low. Fig. 4b shows the phalloidin staining results. The cells in each group stretched well and had abundant pseudopodia; there was no obvious difference in morphology. Fig. 4c shows that more BMSCs are evenly attached to the fibrous membrane under the scanning electron microscope. A larger number of protruding pseudopodia are suitable for cell growth and diffusion, indicating that the fabricated membrane has good cell-trapping ability. Finally, we tested the effect of membranes on cell proliferation using the CCK-8 assay. As shown in Fig. 4d, there was no significant difference (*p* > 0.05) in the proliferation rate of cells in each group compared with the NC group (negative control group) on days 1, 3, and 7 (*p* > 0.05), indicating that the drugs loaded in the membrane had no significant effect on cell proliferation.

### 3.6 Evaluation of the membrane osteogenic capacity in vitro

**Fig. 5:**
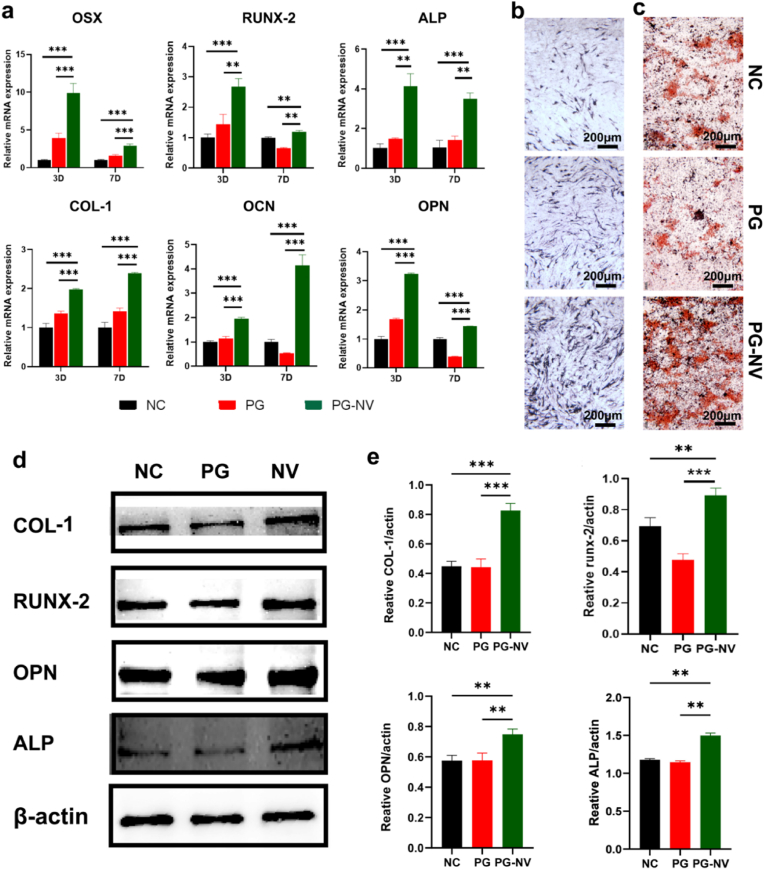
**Osteogenic effect of each group of scaffolds in vitro.** (**a**) Expression of osteogenesis-related genes at 3 and 7 days; (**b**) ALP staining of BMSCs cultured with extracts for 7 days; (**c**) ARS staining of BMSCs cultured with extracts for 14 days; (**d**) Western blotting of COL-1, RUNX-2, OPN, ALP in BMSCs after culturing with the extract for 3 days; (**e**) Quantitative analysis of COL-1, RUNX-2, OPN, ALP compared with GAPDH. Data are shown as mean ± standard deviation. **p < 0.01, ***p < 0.001.

**Fig. 6:**
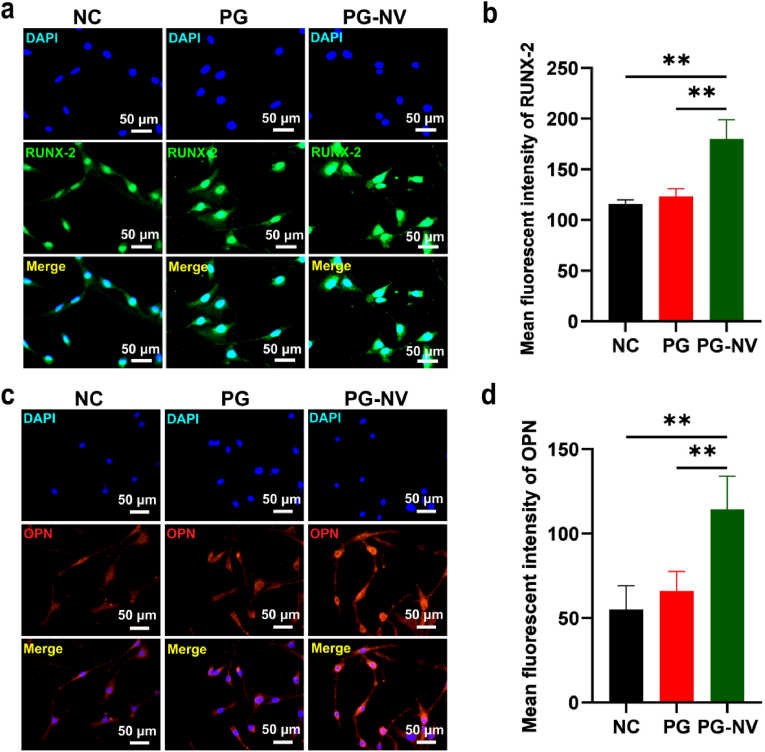
**Immunofluorescent staining of RUNX-2 and OPN in different groups. (a**) RUNX-2; (**b**) Quantitative analysis of fluorescence intensity of RUNX-2; (**c**) OPN; (**d**) Quantitative analysis of fluorescence intensity of OPN. Data are expressed as the mean ± standard deviation (n = 3). **p < 0.01.

To investigate whether the combination of naringenin and vitamin K_2_ promotes bone formation, we detected the expressions of osteogenic-related marker genes in each group on days 3 and 7 using RT-qPCR. As shown in Fig. 5a, on days 3 and 7, the expressions of OSX, RUNX-2, ALP, COL-1, OCN, and OPN genes were remarkably upregulated in the PG-NV group compared with the NC and PG groups, and the differences were statistically significant (*p* < 0.01). However, compared with the NC group, the expressions of osteogenesis-related genes were slightly upregulated and downregulated. It can be concluded that the release of naringenin and vitamin K_2_ of the membrane is the main factor promoting the differentiation of BMSCs toward osteogenesis, whereas the membrane alone has no significant effect on the expressions of osteogenic genes. Fig. 5b shows the results of alkaline phosphatase staining of BMSCs cultured in different membranes for 7 d. Compared with the NC group (control group) and the PG group, the alkaline phosphatase activity of the PG-NV group was the highest. Fig. 5c shows the alizarin red S staining of BMSCs cultured with membranes for 14 d. Compared with the NC group (control group) and the PG group, the PG-NV group had more mineralized calcium nodules, indicating that the PG-NV group had a stronger in vitro mineralization and bone formation ability. Western blotting analysis (Fig. 5d and e) further showed that, compared with the NC group and the PG group, the osteogenesis-related proteins COL-1, RUNX-2, OPN, and ALP expressed in PG-NV were significantly increased (*p* < 0.01). Furthermore, we cocultured BMSCs with membrane extracts for 3 d and performed immunofluorescent staining (Fig. 6). As a result, the expressions of RUNX-2 and OPN in PG-NV were significantly higher than those in NC and PG (*p* < 0.01).

### 3.7 Regulatory effect of membranes on osteoclast growth

Osteoclast formation plays a crucial role in the process of bone growth. We induced RAW264.7 cells toward osteoclasts by using the RANKL.

**Fig. 7:**
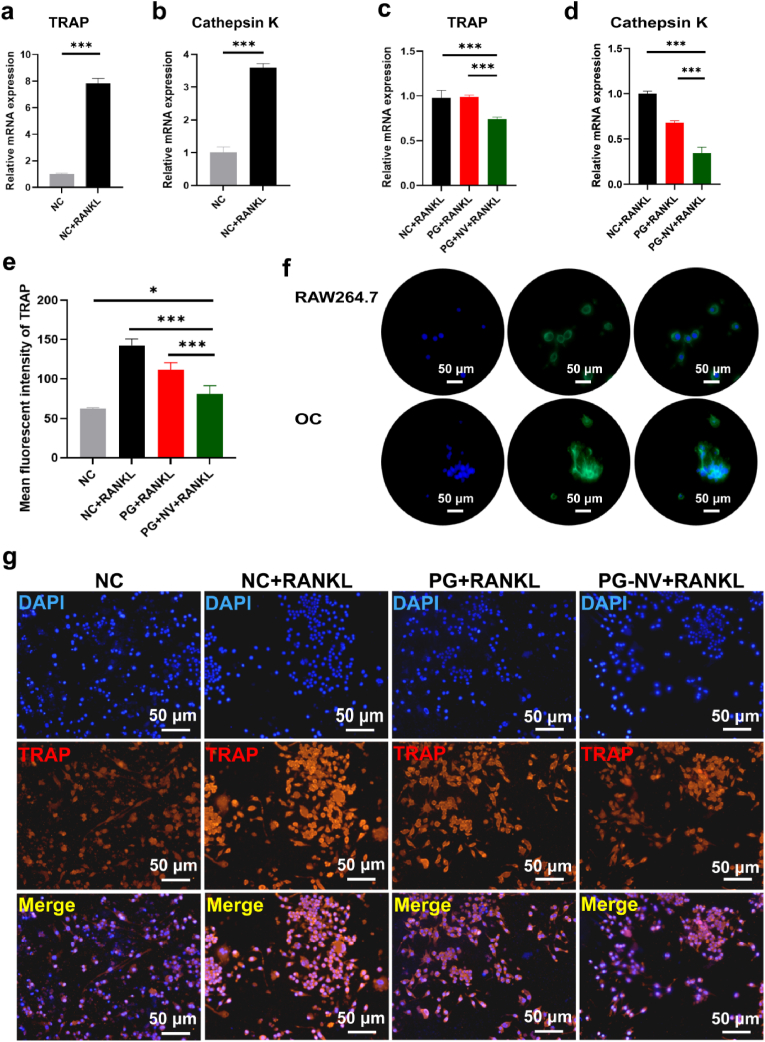
**Effects of different groups of nanofibrous membranes on osteoclasts**. (**a and b**) Changes in the expression levels of osteoclast-related genes TRAP and cathepsin K after induction of RAW264.7 cells using the RANKL; (**c and d**) expression levels of TRAP and cathepsin K in different groups of RAW264.7 after cultured with membranes for five days; (**e**) quantitative analysis of the TRAP fluorescence intensity; (**f**) cytoskeletal staining of RAW264.7 cells and osteoclasts; and (**g**) immunofluorescence staining for TRAP. Data are presented as mean ± SD (*n* = 3). *p < 0.05 and ***p < 0.001.

Subsequently, the RT-qPCR method was used to assess the success of the induction. Osteoclast-associated genes such as TRAP and cathepsin K were significantly upregulated after induction, indicating that RAW264.7 cells successfully transformed into osteoclasts (Fig. 7a–b).

**Fig. 8:**
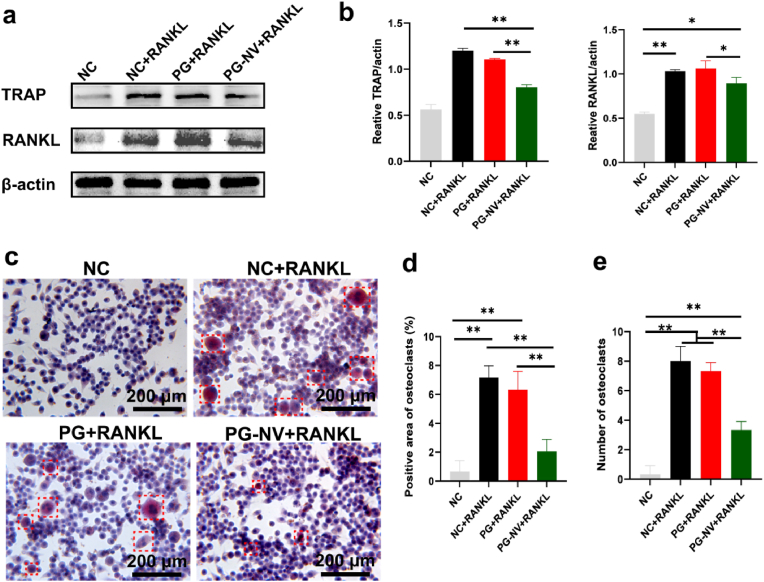
(**A**) Western blotting of TRAP and RANKL; (**b**) quantitative analysis of TRAP and RANKL expressions; and (**c**) TRAP staining of different groups, osteoclasts are circled by a red frame; (**d**) Positive area percentage of osteoclasts; (**e**) The number of osteoclasts in the field of view of a 40× objective lens. Data are presented as mean ± SD (*n* = 3). **p < 0.01.

To study the regulation effect of different membranes on osteoclasts, different scaffolds were cultured with RAW264.7 cells, and the RANKL was added. Five days later, RT-qPCR was performed to detect the expressions of osteoclast-related genes in each group. As shown in Fig. 7c-d, the expressions of TRAP and cathepsin K in the PG-NV + RANKL group were significantly lower than those in the NC + RANKL and PG + RANKL (*p* < 0.001), suggesting that loaded naringenin and vitamin K_2_ inhibited osteoclast formation. In addition, we performed cytoskeleton staining of RAW264.7 cells before and after induction. Fig. 7f shows that the cell morphology changed significantly before and after RAW264.7 cell induction. Before induction, RAW264.7 cells consisted of small, light-colored mononuclear cells that are round or oval. After induction, osteoclasts became large, irregularly shaped multinucleated cells. In addition, using immunofluorescence staining (Fig. 7g), the expression of TRAP protein was found to be the lowest in the NC group and the highest in the NC + RANKL group. The PG-NV + RANKL group was at a moderate level, higher than the NC group (*p* < 0.05), and lower than the NC + RANKL and PG + RANKL groups (*p* < 0.001) (Fig. 7e). These results indicated that naringenin and vitamin K_2_ released from PG-NV membranes had a negative effect on the expressions of osteoclast-associated proteins. Western blot analysis (Fig. 8a–b) showed that the osteoclast-related proteins TRAP and RANKL expressed in the PG-NV + RANKL group were significantly decreased compared with the NC + RANKL and PG + RANKL groups (*p* < 0.01). Finally, TRAP staining was performed. TRAP, an osteoclast marker enzyme, is specifically distributed in osteoclasts and can localize and develop osteoclasts. As shown in Fig. 8c, there were almost no red multinucleated osteoclasts in the NC group, but the proportion of red multinucleated osteoclasts was the largest, with a larger cell volume in the NC + RANKL and PG + RANKL groups. A small amount of red multinucleated osteoclasts were observed in the PG-NV group, but the cells were smaller in size, indicating that the PG-NV membrane inhibited the transformation and growth of osteoclasts to a certain extent.

### 3.8 Evaluation of the new bone growth of the membrane in vivo

A rat calvarial bone defect model was used to study the ability of nanofibrous membranes loaded with naringenin and vitamin K_2_ to promote osteogenesis and regulate osteoclast growth in vivo. At 4 and 8 weeks after the operation, rat skull specimens were collected and formalin-fixed.

### 3.9 Micro-CT analysis

**Fig. 9:**
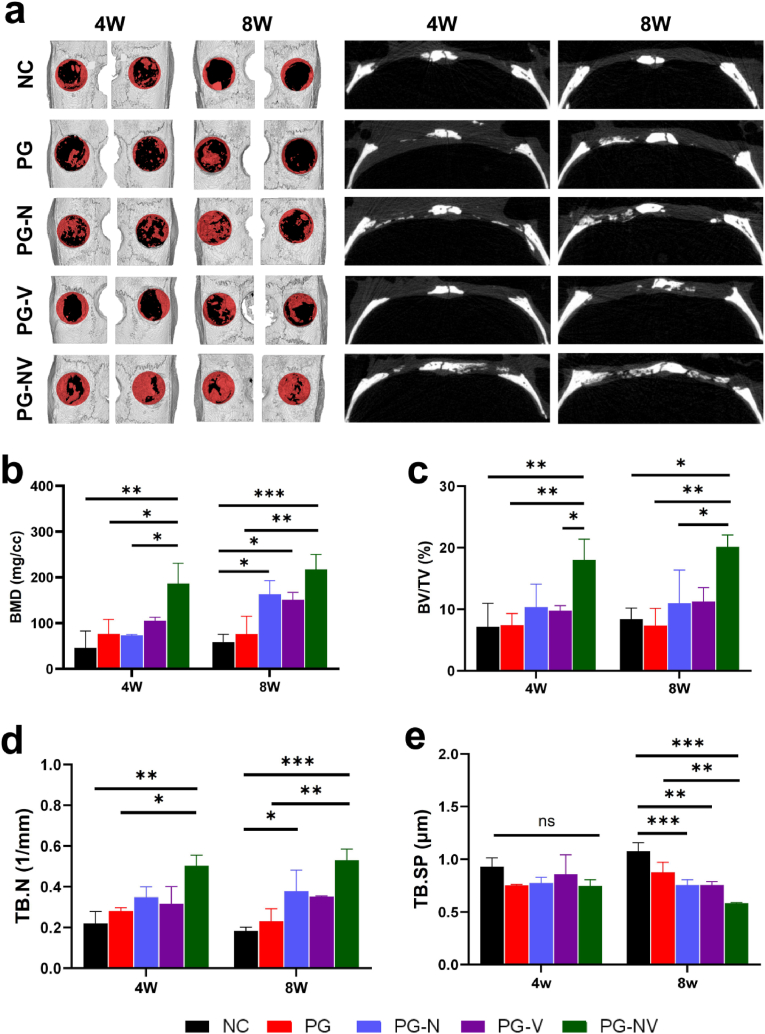
**Micro-CT.** (**a**) Three-dimensional and sagittal images of rat skull defects at four and eight weeks postoperatively. The red in the circle represents new bone, and the black represents bone defect; (**b**) bone mineral density (BMD), (**c**) bone volume/total volume (BV/TV), (**d**) number of trabeculae (TB.N), and (**e**) trabecular separation (TB.SP). Data are presented as the mean ± SD (*n* = 3). *p < 0.05 and **p < 0.01.

After 4 and 8 weeks of membrane implantation, the new bone growth of each group of fibrous membranes was evaluated using micro-CT. As shown in Fig. 9a, the fibrous membrane was slowly degraded and gradually replaced by new bone in rats. After four weeks of membrane implantation, compared with other groups, the PG-NV group had a large amount of new bone formation at the bone defect, and the new bone tissue formed in the PG-N and PG-V groups was more than that in the NC and PG groups. There was only a small amount of new bone formation in the bone defect in the NC and PG groups. At eight weeks postoperation, the new bone growth trend in each group was similar to that at four weeks postoperation. The new bone tissue in the PG-NV group almost filled the bone defect, and a large amount of new bone was also found in the center of the bone defect in the PG-N and PG-V groups. We performed subsequent quantitative analysis of osteogenesis-related indicators (Fig. 9b–e). Overall, the bone mineral density, bone volume fraction, and trabecular number in the PG-NV group were significantly higher than those in the other four groups (*p* < 0.05). Moreover, the bone density, bone volume fraction, and bone trabecular number of PG-N and PG-V were higher than those of the NC and PG groups. The degree of trabecular separation in the PG-NV group was lower than in the NC and PG groups (*P* < 0.05).

### 3.10 Histological evaluation of bone regeneration

**Fig. 10:**
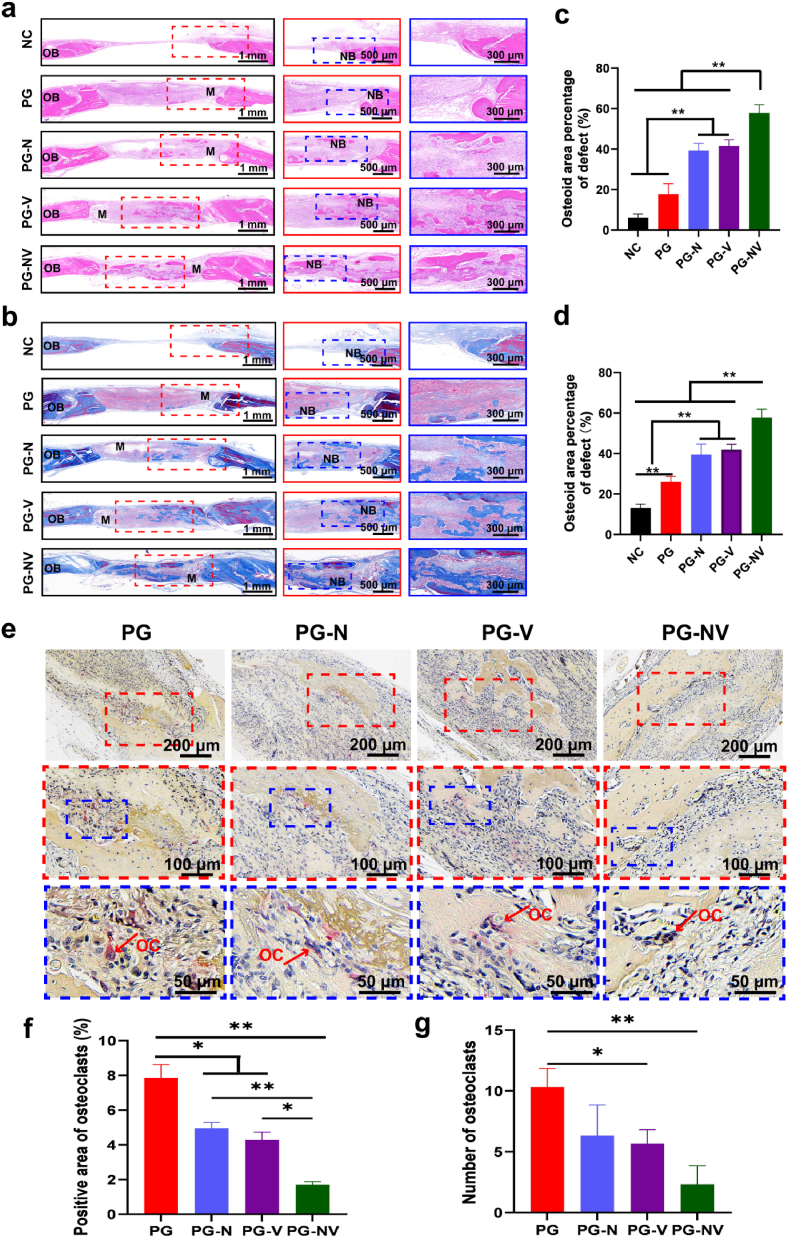
**Histological staining of different groups at 8 weeks postsurgery**. (**a**) Hematoxylin and eosin staining and (**b**) Masson's trichrome staining. (**c and d**) Quantitative analysis of new bone area using hematoxylin and eosin (**c**) and Masson's trichrome (**d**) staining. (**e**) TRAP staining in different groups at four weeks postoperatively. Arrows point to osteoclasts. (**f**) Percentage of osteoclasts in the total area under a 120X field of view. (**g**) The number of osteoclasts under a 50X field of view. Data are shown as the mean ± SD (*n* = 3). **p < 0.01. Abbreviations: NB, new bone; OB, host bone; and M, membrane.

Hematoxylin and eosin staining and Masson's trichrome staining were performed on the skull specimens eight weeks postsurgery. As shown in Fig. 10a-b, the defect in the NC group was filled with a large amount of connective tissue, and the PG group had a large amount of connective tissue infiltration but less new bone tissue growth. The PG-N and the PG-V groups had the smaller new bone growth at the edge and center of the bone defect, whereas the PG-NV group had the most new ingrown hard tissue, covering almost the entire bone defect. Subsequently, we quantified the new bone tissue (Fig. 10c–d). The results showed that the PG-N and PG-V groups stimulated new bone growth, and the collagen deposition was higher than that of the NC and PG groups (*p* < 0.01). Compared with the PG-N and PG-V groups, the ability of the PG-NV group to stimulate osteogenesis and collagen deposition was significantly increased (*p* < 0.01). TRAP staining was used to evaluate the growth regulation effect of membranes on osteoclasts. Fig. 10e shows the TRAP staining of sections in each group at four weeks postoperatively. Osteoclasts were stained wine red. The PG group had the most osteoclasts around the new bone. The number of osteoclasts decreased in the PG-N and PG-V groups, and the number of osteoclasts was the least in the PV-NV group.

### 3.11 Immunohistochemical evaluation

**Fig. 11:**
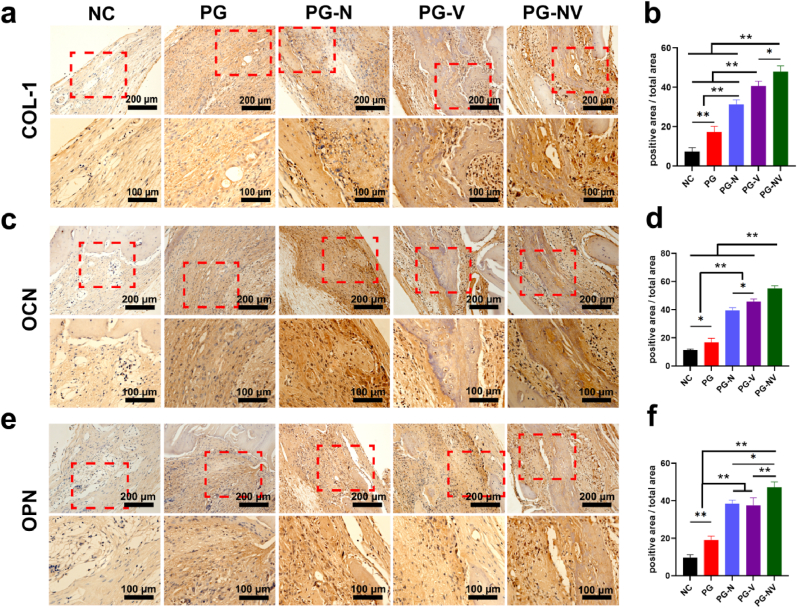
**Immunohistochemical staining at 8 weeks post-surgery.** (**a**) Type I collagen (COL1), (**c**) osteocalcin (OCN), and (**e**) osteopontin (OPN) in the bone defects at eight weeks post-surgery. (**b**) Quantitative analysis of COL-1, (**d**) OCN, and (**f**) OPN. Data are shown as the mean ± SD (*n* = 3). *p < 0.05 and **p < 0.01.

Immunohistochemical staining was performed on the samples eight weeks postsurgery. Fig. 11 shows the expressions of osteogenesis-related proteins (RUNX-2, OCN, and OPN) in each component. Compared with other groups, the expressions of RUNX-2, OCN, and OPN in the PG-NV group were the highest (*p* < 0.05). Compared with the NC and PG groups, the expressions of RUNX-2, OCN, and OPN in PG-N and PG-V groups were also significantly increased (*p* < 0.05). Therefore, it can be concluded that the PG-NV group has the best bone regeneration ability. Both naringenin and vitamin K_2_ can promote the growth of new bone and synergistically affect bone regeneration.

## 4 Discussion

Countless patients have huge bone defects worldwide every year. These bone defects are difficult to heal due to trauma, infection, osteoporosis, and tumors [25]. Therefore, searching for ideal bone implant materials has become a research hotspot in bone tissue engineering. An ideal bone implant material should meet the following conditions [8,9,26]: 1. The implant has good biocompatibility and no or low cytotoxicity; 2. Excellent mechanical properties and plasticity; 3. Good pore structure promoting the growth of bone tissue; 4. A degradation rate corresponding to the growth of bone tissue; and 5. Osteoinductive function. It has been more than 80 years since Formals invented electrospinning technology. With the continuous development of this technology, the nanofibrous material prepared now has high porosity, a high specific surface area, and a structure similar to the extracellular matrix, which can promote cell growth and its physiological process [27].

Given the shortcomings of PCL as a material for bone tissue engineering, it is a very promising method to combine PCL with other bioactive materials to prepare composite scaffolds and improve their degradability, hydrophilicity, and osteoinductivity [28]. Bahcecioglu [29] incorporated PCL into the agarose/GelMA hydrogel scaffold using 3D printing, and the meniscus scaffold thus constructed can replace the full meniscus. The composite material improves the compressive strength of the hydrogel scaffold and is more suitable for the growth and proliferation of cells. In this study, both gelatin and PCL were used as base materials to make up for the deficiency of both. The results show that the nanofibrous membrane prepared using this method has excellent performance in terms of biocompatibility, hydrophilicity, mechanical properties, and degradation rate. Ma Rui [30] divided the titanium mesh scaffolds into a low porosity group of 55%, a medium porosity group of 62%, and a high porosity group of 68%. The results showed that within a specific range, the greater the porosity, the better the bone growth. Inspired by this, we covered a copper mesh on the electrospun collection device and used layered spraying technology to make the constructed membrane have a unique mesh-like sandwich structure. The copper mesh can change the electric field, changing the distribution of nanofibers. Therefore, in the center of the mesh, the nanofibers are sparsely distributed, resulting in a thin membrane and large porosity in this area, which is conducive to the growth of new bone tissue. However, on the mesh line of the nanofibrous membrane, the nanofibers are densely distributed, resulting in a thick membrane and low porosity in this area, which is conducive to improving the membrane's mechanical properties. Viewed from the cross-section, the membrane has the characteristics of a dense shell and loose core fibers. Hassanin [31] et al. showed that the drug release rate in the middle layer of the three-layer sandwich structure was greatly slowed down. In addition, Chaparrp [32] et al. changed the pore size and porosity of tubular polyglycolide by sintering to change the drug release rate and found that the denser the structure, the slower the drug release rate. It indicated that the electrospun nanofibers prepared with these two structures realize the slow release of drugs and promote bone tissue growth.

Biocompatibility assessment of bone tissue engineering is an important tool to investigate whether materials can provide a good microenvironment for cell growth and proliferation [33]. Especially for drug-loaded scaffolds, the sudden release of a large number of drugs in the body increases the toxicity of the drug and damages human health while reducing the utilization rate of the drug. Ideally, the drug concentration should be maintained between the minimum effective concentration and the toxic concentration for a long time [11,34,35]. Therefore, more complex electrospun structures, such as coaxial electrospinning, triaxial electrospinning, and multi-layer sandwich electrospinning structure, were used for drug loading. These special structures can change the drug distribution position, better control the drug release rate, improve drug utilization, and reduce drug side effects [10]. In this study, approaches such as live/dead staining, cytoskeleton staining, CCK-8, and SEM were used to observe the number and morphology of cells on the surface of the membrane. It was found that BMSCs grow well on the nanofibrous membrane, which has good biocompatibility and can provide the required microenvironment for the growth, proliferation, and differentiation of cells.

A scaffold material for bone implants must have suitable physical and chemical properties and good biocompatibility, aiming to promote the repair of bone defects [36,37]. Early electrospun membranes mainly used single or compound solutions, including Polycaprolactone, sodium alginate, silk fibroin, polyvinyl alcohol, and chitosan. The electrospun membranes fabricated using this method have poor mechanical properties and no osteoinductive ability. An implant for bone defect has great limitations [38]. Therefore, some researchers tried to endow the membrane with osteogenesis, antibacterial, anti-inflammatory, and angiogenesis functions by doping or adsorbing drugs or ions or combining peptides and proteins on the implant. It has been reported that incorporating nano-hydroxyapatite into polymer membranes such as polylactic acid and Polycaprolactone can enhance their mechanical strength and thermal stability while promoting cell attachment and proliferation on the membrane surface [39,40]. Abazari MF [41] et al. prepared functional nanofibrous membranes by mixing basic fibroblast growth factor with polycaprolactone/polyvinylidene fluoride and proved that membranes containing basic fibroblast growth factor could make pluripotent stem cells more viable, mineralized, and differentiated. Ma [42] mixed hydroxyapatite and attapulgite into the 3D printing scaffold. The strong combination of the two makes the constructed scaffold better in promoting bone formation. Simultaneously, the attapulgite can promote the growth of blood vessels, endowing the scaffold with multiple functions. Inspired, we loaded naringenin and vitamin K_2_ into the fibrous membranes. Naringenin can enhance the osteoblast differentiation and mineralization ability of hPDLSCs and rBMSCs by activating Runt-related transcription factors, BMP-2, and alkaline phosphatase and promote the repair of bone defects. Naringenin also has the effect of treating osteoporosis. It inhibits osteoclast formation and reduces bone resorption by reducing the expressions of TRAP and protein kinase K (cathepsin K) [19,[43], [44], [45]]. Vitamin K_2_ can promote the growth and differentiation of osteoblasts, promote the deposition of bone calcium, and treat osteoporosis [22,24]. Clinical research shows that it can improve bone microstructure, increase bone mineral ratio, increase bone density, prevent excessive bone loss, and reduce the risk of osteoporotic fractures and the possibility of osteoporosis in postmenopausal women taking vitamin K2 therapy. In addition, studies have shown that a daily intake of more than 45 mg of vitamin K_2_ can effectively improve osteoporosis in postmenopausal women, which is much higher than the daily intake of humans, but there is no obvious toxic effect, indicating that vitamin K_2_ is a relatively safe drug for the human body [46]. Regarding the osteogenesis of nanofiber membrane, we detected osteogenesis-related genes (OSX, RUNX-2, ALP, COL-1, OPN, and OCN) and osteogenesis-related factors (COL-1, RUNX-2, OPN, and ALP) expression levels of membranes loaded with naringenin and vitamin K_2_ were significantly upregulated. In subsequent ALP and alizarin red S staining, the PG-NV group had the most calcium nodules and stronger mineralization ability than the NC and PG groups. These results showed that the PG-NV group positively affects bone growth. In terms of membranes inhibiting the growth of osteoclasts, RT-qPCR detection showed that RANKL, cathepsin K, and other osteoclast-related genes were significantly decreased under the action of naringenin and vitamin K_2_. In the western blotting experiment, the expressions of osteoclast-associated proteins (RANKL and TRAP) decreased compared with NC and PG groups. In TRAP staining, the number of osteoclasts induced in the PG-NV + RANKL group was significantly decreased compared with NC + RANKL and PG + RANKL groups. Based on the above results, under the action of naringenin and vitamin K_2_, the transformation of RAW264.7 cells into osteoclasts could be inhibited, and the ability of RANKL to induce osteoclasts could be weakened.

In animal experiments, micro-CT, HE, and Masson trichrome staining showed a large amount of new bone tissue in the bone defect in the PG-NV group, and the bone-promoting ability of this group was significantly better than that of others. PG-N and PG-V groups have stronger bone defect repair ability than the PG and NC groups. TRAP staining was performed on the sections of each group, and the results showed that both naringenin and vitamin K_2_ could inhibit the formation of osteoclasts, and the combined effect of the two was stronger. The subsequent immunohistochemical staining showed that the protein expression levels of OCN, COL-1, and OPN in the PG-NV group were significantly higher than in other groups. The results of in vivo animal experiments also showed that naringenin and vitamin K_2_ have a synergistic effect on osteogenesis, which can enhance the ability of the membrane to induce osteogenesis. Naringenin and vitamin K2 have broad application prospects in bone tissue engineering, especially in patients with osteoporotic bone defects.

## 5 Conclusion

In this study, we used electrospinning technology to construct nanofibrous membranes loaded with naringenin/vitamin K_2_ with mesh and sandwich structures for bone defect repair. The results of cell experiments showed that the fiber membrane had good cell compatibility and could provide a good microenvironment for cell growth, proliferation, and differentiation. Nanofibrous membranes incorporated with naringenin and vitamin K_2_ can promote the osteogenic differentiation of BMSCs by increasing the expressions of OSX, RUNX-2, ALP, COL-1, OCN, and OPN genes, while it also inhibited the expressions of TRAP and cathepsin K genes, and reduced the formation of osteoclasts. In the postoperative rat skull defect model, micro-CT, histology, and immunohistochemical staining showed that the PG-NV group had the largest amount of new bone and the strongest ability to repair bone defects. In summary, the ability of membranes in each group to promote bone growth was PG-NV group > PG-N group, PG-V group > PG group. Naringenin and vitamin K_2_ can synergistically promote bone formation, regulate osteoclasts, and have good application prospects in bone tissue engineering.

## Credit author statement

Jiafeng Wang: Methodology, Formal analysis, and Writing – Original Draft, Longhui Shao Methodology, Formal analysis, Xiaoyu Wu: Date analysis，Data curation, Chun Liu: Writing – Review & Editing, Su Ni: Methodology，software, Ting Dai: Formal analysis, Validation, Hongwei Liu: Funding acquisition, Supervision, Hongbin Zhao Conceptualization, Resources，Project administration.

## Declaration of competing interest

The authors declare that they have no known competing financial interests or personal relationships that could have appeared to influence the work reported in this paper.

## Data Availability

No data was used for the research described in the article.
