# Supplementary material for: Electrospun sandwich mesh structures loaded with naringenin and vitamin K2 polycaprolactone/gelatin nanofibers synergistically promote bone regeneration
Source: Mater Today Bio. 2023 Sep 15;23:100794. doi: 10.1016/j.mtbio.2023.100794 (PMC10520447; doi:10.1016/j.mtbio.2023.100794)
Supplement: Multimedia component 1 [file mmc1.docx]

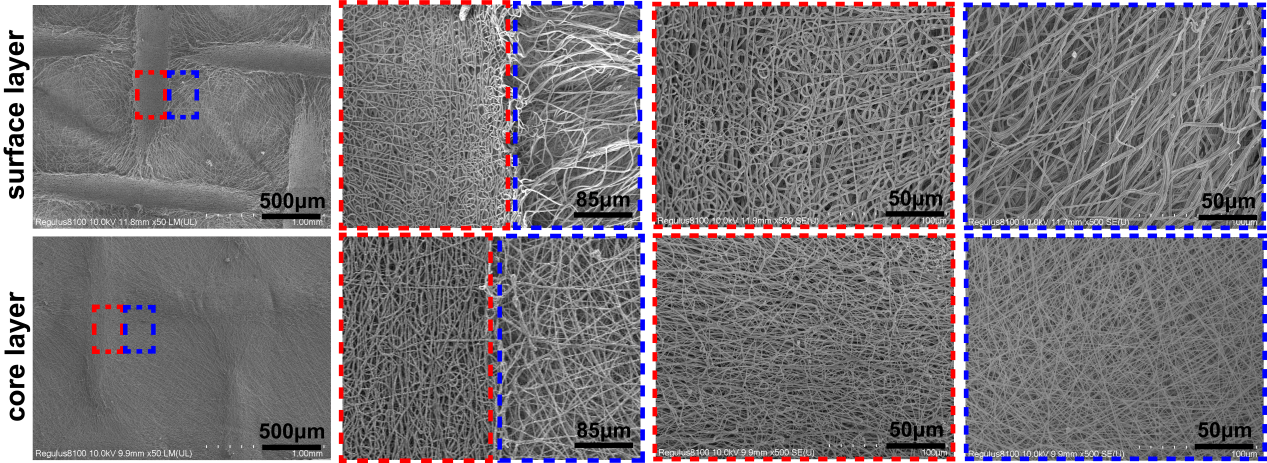


**Figure.S1 SEM of the interface between the surface and core layers.**


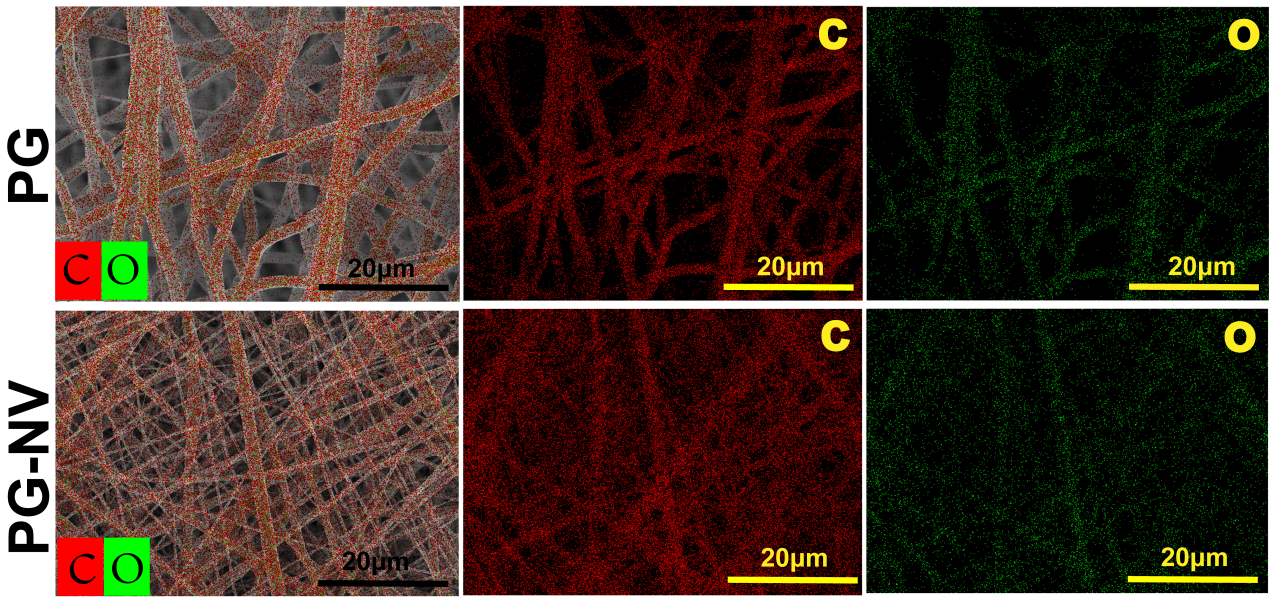
**Figure.S2 Element mapping for PG groups and PG-NV groups.**


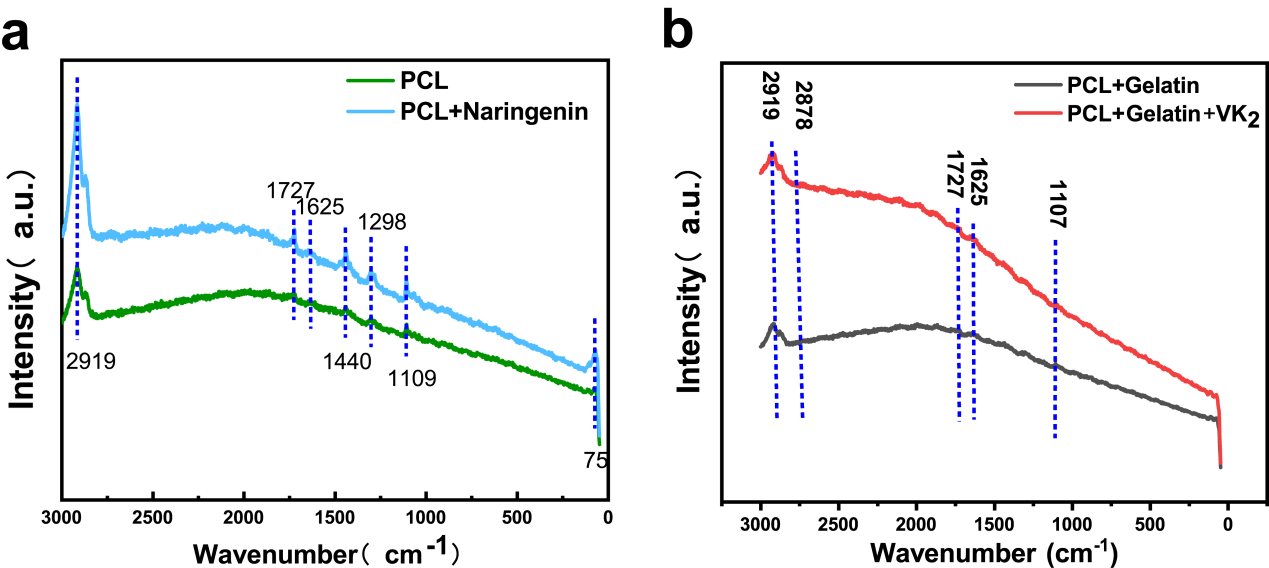


**Figure.S3 Raman spectroscopy.** (**a**) Raman spectra of PCL and PCL+naringenin; (**b**) Raman spectra of PCL+Gelatin and PCL+Gelatin+VK_2_.
